# Prognostic significance of non-sustained ventricular tachycardia on stored electrograms in pacemaker recipients

**DOI:** 10.1371/journal.pone.0225059

**Published:** 2019-11-15

**Authors:** Gianluigi Bencardino, Francesco Raffaele Spera, Gaetano Pinnacchio, Francesco Perna, Maria Lucia Narducci, Gianluca Comerci, Gemma Pelargonio, Francesca Augusta Gabrielli, Giulio La Rosa, Gaetano Antonio Lanza, Filippo Crea

**Affiliations:** 1 Dipartimento di Scienze Cardiovascolari e Toraciche, Fondazione Policlinico Universitario Agostino Gemelli IRCCS, Roma, Italy; 2 Università Cattolica del Sacro Cuore, Roma, Italy; University of Bologna, ITALY

## Abstract

**Background:**

Little is known about the prognostic significance of non-sustained ventricular tachycardia (NS-VT) in outpatients scheduled for routine pacemaker controls. We therefore sought to investigate the prognostic significance of non-sustained ventricular tachycardia on stored electrograms in pacemaker recipients.

**Methods:**

We enrolled patients implanted with dual chamber pacemaker for atrioventricular block or sinus node dysfunction from 2010 to 2016, with LVEF> 45%, older than 18 years, with at least 3 device interrogations at follow-up. Data were collected about medical history, pharmacological therapy at implantation, pacemaker programming, NS-VT occurrence, long-term survival.

**Results:**

A total of 308 patients were included in the final analysis, with median follow-up time of 56 months. No ventricular arrhythmic episodes were documented in 221 patients (Group 1), whereas 87 had at least 1 episode of NS-VT during follow-up (Group 2). As a whole, 282 episodes of NS-VT were documented. There was a higher prevalence of previous myocardial infarction and slightly lower left ventricular ejection fraction (LVEF) in Group 2. The primary endpoint (all-cause mortality) occurred in 50 patients (22%) of Group 1 and 12 (14%) patients of Group 2 (p = 0.07). Clinical predictors of all-cause mortality at univariate analysis included age, LVEF and coronary artery disease (CAD). Only age and CAD, however, remained as predictors of mortality at multivariable analysis. A sizeable, but not statistically significant, portion of patients who died had a de novo occurrence of NS-VT at the last pacemaker check.

**Conclusion:**

Our data do not support a prognostic role for the detection of NS-VT during pacemaker controls.

## Introduction

Pacemaker technology has evolved since the first pacemaker implantation over 50 years ago [1]. Permanent pacemakers detect and store both atrial and ventricular arrhythmias with a high degree of sensitivity. Supraventricular and ventricular arrhythmias are commonly detected during routine pacemaker controls in clinical practice, but their significance and prognostic implications are currently unclear. While information regarding the incidence and significance of non-sustained ventricular tachycardia (NS-VT) has been reported in patients with reduced left ventricular ejection fraction (LVEF) [2,3], with implanted defibrillators [4], in the setting of acute myocardial infarctions [5], and in patients with coronary artery disease (CAD) who underwent coronary revascularization [6], little is known about the prognostic significance of NS-VT in outpatients scheduled for routine pacemaker controls. Prior studies that examined NS-VT in outpatients without evidence of heart failure [7] or a history of cardiovascular disease [8] used 24-hour ambulatory electrocardiographic monitoring. In further 24-hour recordings, however, NS-VT is only reproducible in half of these patients [8,9], however, a fact that complicates the interpretation of the finding. Yet, establishing the prognostic significance of NS-VT in the outpatient setting has important implications for clinical management. Therefore, we examined a cohort of patients without or with NS-VT identified on stored electrograms from permanent dual-chamber pacemakers and compared the clinical characteristics and long-term outcome of the two groups.

## Methods

### Study subjects

**Fig 1:**
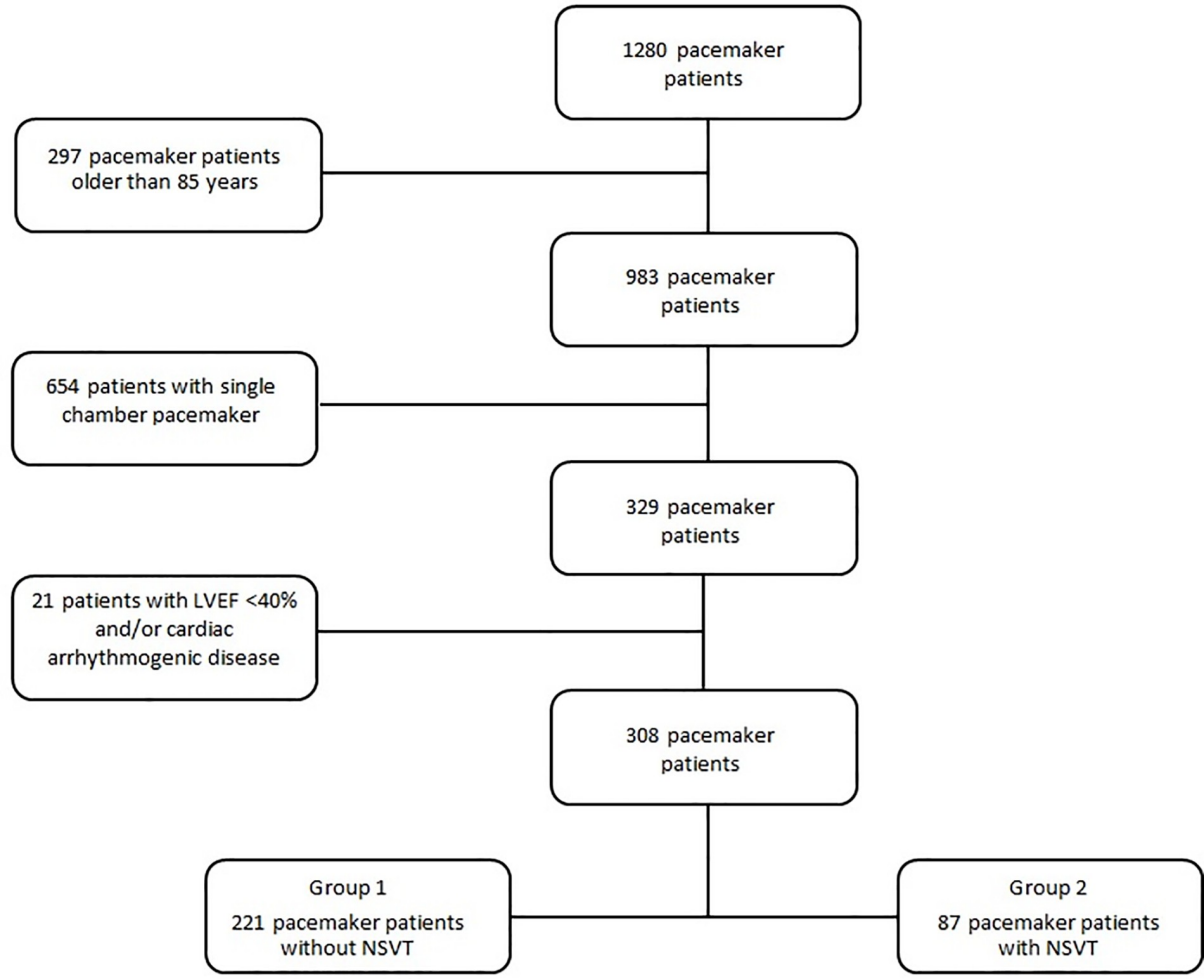
Study flowchart. Flowchart illustrating the distribution of pacemaker patients in data analysis after the application of exclusion criteria. LVEF = Left Ventricular Ejection fraction; NS-VT = non sustained ventricular tachycardia.

We enrolled 308 consecutive patients (mean age 72 ± 12 years) admitted to our Department of Cardiology from 2010 to 2016 who underwent dual chamber pacemaker implantation because of guideline-accepted indications and fulfilled the following inclusion criteria: 1) age between 18 and 85; 2) at least 3 device interrogations following their initial implant. Exclusion criteria included depressed left ventricular function (i.e. LVEF ≤45%) and overt arrhythmogenic cardiac diseases. In addition, patients with single-chamber devices were excluded due to possible concerns about reliability of differential diagnosis between ventricular and supraventricular arrhythmic events (Fig 1).

The lower atrial pacing rate was programmed at 60–70 bpm and the upper tracking rate at 110–120 bpm; the rate response function was activated in all patients with sinus node disease; furthermore, in order to minimize right ventricular pacing, an AAI-DDD algorithm was activated whenever available; otherwise, AV interval was delayed to 300 ms.

Inpatient and outpatient medical records were reviewed to obtain demographic information, including age, gender, cardiovascular risk factors, symptoms suggestive of arrhythmias, history of cardiovascular disease, past medical history and medications, along with electrocardiograms, transthoracic echocardiograms, nuclear stress tests and cardiac catheterization (when available). Hypertension was defined as blood pressure >140/90 mmHg or consumption of anti-hypertensive drugs; hypercholesterolemia was defined as blood cholesterol levels >200 mg/dL or consumption of anti-cholesterolemic drugs; diabetes was defined as fasting glucose blood levels >126 mg/dL or consumption of anti-diabetic drugs. CAD was diagnosed in case of a documented history of previous myocardial infarction (MI), percutaneous and/or surgical coronary intervention, documented myocardial ischemia at non-invasive tests and/or coronary stenoses at angiography. Patients were considered to have valvular heart disease if they met standard definitions of severe aortic and/or mitral stenosis or regurgitation as assessed by echocardiography [10, 11] Other cardiovascular disorders were identified according to documented evidence.

Informed consent for data collection was obtained from each patient. The study protocol conformed to the ethical guidelines of the Declaration of Helsinki and was approved by the institutional review board of the University Hospital Fondazione Policlinico A. Gemelli IRCCS, Rome (Italy).

### Follow up and end-point

Patients were scheduled for device interrogation at 6-month intervals. At the time of each visit, data concerning pacemaker function, percentage of pacing, arrhythmic episodes, as well as clinical data, were recorded. Device recordings were analyzed by an expert electrophysiologist who revised and classified the electrograms of every tachyarrhythmic episode in a blind way with regard to clinical data. The percentage of right ventricular (RV) pacing was determined from the latest device interrogation in the study period. NS-VT was defined as 3 or more consecutive beats of tachycardia with an RR interval of < 600 ms (> 100 beats/min) and lasting less than 30 seconds.[12] Ventricular tachyarrhythmias were individually verified by confirming either atrioventricular dissociation and/or change in electrogram morphology not thought to be secondary to aberrancy. The NS-VT burden was defined as the number of episodes of recorded NS-VT throughout the individuals’ follow up period (the time from implant to the last device interrogation).

The primary endpoint, death from any cause, was ascertained by telephone calls to the family or by examination of hospital records.

### Statistical analysis

The distribution of variables was assessed by Kolmogorov–Smirnov test. Continuous variables were compared by analysis of variance (ANOVA) or Kruskal–Wallis test, as indicated. In case of global statistical significance, multiple between-group comparisons were done by unpaired *t*-test or Mann–Whitney test, respectively. Chi-square test was used to compare categorical variables. Bonferroni correction of statistical results was always applied for multiple comparisons. Follow-up time was calculated as the interval from time of implant to time of death or last follow-up. Kaplan–Meier survival curves were constructed for the estimation of unadjusted survival distributions among groups as well as for subgroup analyses. Log-rank tests were used to compare survival curves of the 2 groups. Univariate Cox regression analysis was applied to assess the association with death of ventricular arrhythmic episodes, as well as of clinical and laboratory variables including age, hypertension, diabetes mellitus, LVEF, presence of chronic kidney disease and presence of coronary artery disease. A multivariable Cox regression analysis was then applied to identify prognostic variables independently associated with mortality. To this aim, only variables with a p*<*0.1 at univariate analysis were included in the multivariable model. Analyses were carried out by the SPSS 20.0 statistical software (SPSS Italia, Florence, Italy). Data are reported as mean ± deviation standard. Statistical significance was considered for 2-sided p*<*0.05.

## Results

A total of 308 patients were included in the study. The cause of pacemaker implantation was advanced atrioventricular block in 160 patients (52%) and sinus node disease in 148 patients (48%). Overall, the patient population included 78 (25%) patients with preexisting CAD and 42 patients (13.6%) with valvular heart disease. Mean LVEF was 57±6%; 64 (20.8%) patients had some evidence of renal failure (glomerular filtration rate *<*60 mL/min). With respect to medical therapy, 142 patients (46%) were taking *β*-blockers and 86 (28%) angiotensin converting enzyme inhibitors; overall, 27 patients were taking antiarrhythmic medications, either amiodarone (n = 13) or a class IC antiarrhythmic drug (either propafenone or flecainide, n = 14). The median follow-up time was 56 months (range 14–110).

Of the 308 patients included in the study, no ventricular arrhythmic episodes were documented in 221 patients (72%) (Group 1), whereas 87 (28%) had at least 1 episode of NS-VT during follow-up (Group 2). On the whole, 282 episodes of NS-VT (mean length 11 beats, cycle length 349±26 ms) were documented. Baseline clinical characteristics of patients with and without NS-VT episodes are summarized in **Table 1**. As shown, the 2 groups were similar for most variables with the exception of a higher prevalence of previous myocardial infarction (10.8% vs. 4.4%, p = 0.039) as well as a slightly lower LVEF (55±9% vs. 58±8%, p = 0.023) in patients with NS-VT. There were no significant differences between the 2 groups with regard to pharmacological therapy, renal failure or prevalence of paroxysmal atrial fibrillation.

**Table 1:** Comparison of clinical characteristics of subjects with and without NS-VT.

	Pts without NS-VT (221)	Pts with NS-VT (87)	P Value
Age (years)	71.6± 11	72.9± 9	0.35
Gender (M/F)	135/86	59/28	0.27
Renal dysfunction (%)	53 (24%)	22 (25.3%)	0.90
Atrial fibrillation(%)	75 (33.9%)	25 (28.7%)	0.34
Chronic total occlusion (%)	8 (3.6%)	3 (3.4%)	0.93
Previous ACS (%)	9 (4%)	9 (10.3%)	**0.039**
Valvular heart disease (%)	33 (14.9%)	13 (14.9%)	0.91
LVEF (%)± SD	57.8± 8	55.1± 9	**0.023**
LVEF<50% (%)	24 (10.9%)	17 (19.5%)	0.061
Previous cardiac surgery (%)	38 (17.1%)	12 (13.8%)	0.40
TAVI (%)	8 (3.6%)	2 (2.3%)	0.53
Beta-blocker (%)	107 (48.4%)	44 (50.6%)	0.84
Amiodarone (%)	12 (5.9%)	4 (4.1%)	0.59
Class IC drugs	13 (6.4%)	4 (4.1%)	0.60
ACE inhibitor or ARB (%)	118 (53.4%)	46 (52.9%)	0.80

ACE = Angiotensin-converting enzyme; ACS = acute coronary syndrome; ARB = angiotensin receptor blocker; LVEF = left ventricular ejection fraction; NS-VT = non sustained ventricular tachycardia.

**Fig 2:**
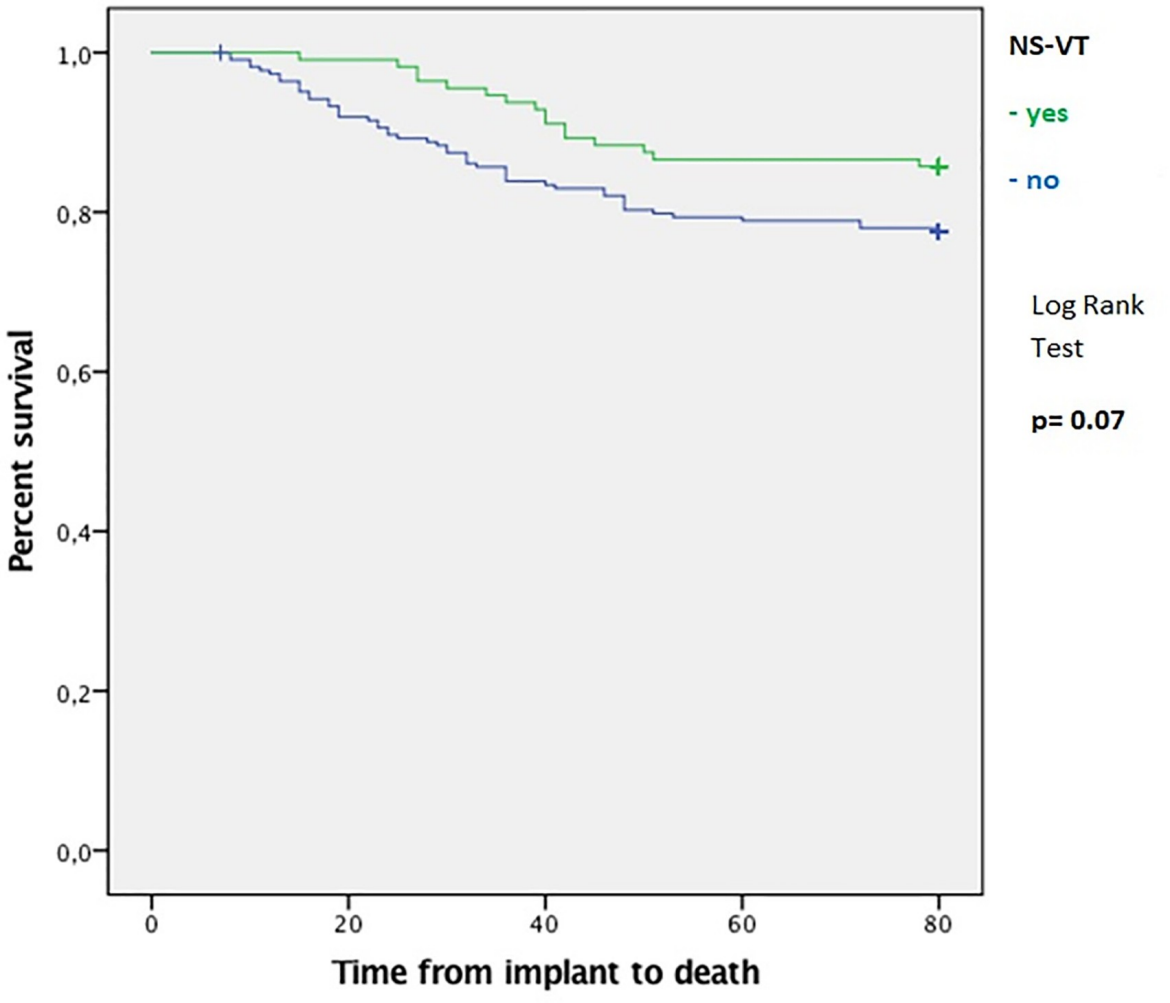
Cumulative survival at follow-up. Kaplan-Meier analysis of survival according to the occurrence of NS-VT on stored electrograms in pacemaker recipients. NS-VT = non sustained ventricular tachycardia.

At the end of follow-up, the primary endpoint (all-cause mortality) occurred in 50 patients (22%) in Group 1 and in 12 patients (14%) in Group 2 (HR, 0.59; 95% CI 0.15–1.10; p = 0.078). The average time from implantation to death was 69±1.5 vs. 74±1.4 months in Group 1 and Group 2, respectively (Fig 2). Among Group 2 patients, time from implantation to the first NS-VT episode was 17±5 months, whereas time from NS-VT to death was 13.2±10 months.

The causes of death were also similar in the 2 groups. Specifically, death was caused by non-cardiovascular comorbidities in 27 patients (54%) of group 1 and 7 patients (58.4%) of group 2 (p = 0.86), by acute heart failure in 15 (30%) and 3 (25%) patients of the 2 groups, respectively (p = 0.75) and by non-arrhythmic infarct-related complications in 4 (8%) and 1 (8.3%) patients of the 2 groups, respectively (p = 0.97). The cause of death could not be ascertained in 3 patients of group 1 (1.4%) and 1 patient (1.1%) of group 2.

Variables associated with all-cause mortality at univariate survival Cox regression included age (p *=* 0.002), LVEF (p = 0.02) and a history of CAD (p = 0.042) **(Table 2)**. Only age (p = 0.006) and LVEF (p = 0.018), however, maintained a significant association with death at multivariable analysis **(Table 3)**.

**Table 2:** Univariate association of variable with the primary end-point of all-cause mortality.

	HR (95% CI)	P value
Age	1.06 (1.02–1.10)	**0.002**
Gender (male vs female)	0.86 (0.51–1.45)	0.57
Valvular heart disease	0.88 (0.42–1.87)	0.75
Previous ACS	1.53 (0.61–3.84)	0.36
Previous cardiac surgery	1.09 (0.55–2.17)	0.80
TAVI	1.03 (0.25–4.22)	0.97
Renal dysfunction	1.15 (0.64–2.04)	0.64
LVEF	0.96 (0.93–0.98)	**0.002**
Beta-blocker	1.693 (0.98–2.92)	0.058
Amiodarone	0.77 (0.30–1.03)	0.58
ACE inhibitor or ARB	1.04 (0.6–1.76)	0.90
Atrial fibrillation	1.02 (0.6–1.73)	0.93
NS-VT episodes	0.59 (0.15–1.1)	0.078
NS-VT episodes at last FUP	2.11 (0.96–4.64)	**0.064**

ACE = Angiotensin-converting enzyme; ACS = acute coronary syndrome; ARB = angiotensin receptor blocker; CI = Confidence interval; FUP = follow-up; HR = Hazard ratio; LVEF = Left ventricular ejection fraction; NS-VT = non sustained ventricular tachycardia.

**Table 3:** Predictors of mortality at multivariate Cox regression analysis.

	HR (95% CI)	P value
Age	1.06 (1.01–1.11)	**0.006**
LVEF	0.97 (0.94–0.99)	**0.018**
NS-VT episodes	0.45 (0.24–1.24)	0.071
NS-VT episode at last FUP	1.58 (0.56–4.43)	0.38

CI = Confidence interval; FUP = follow-up; HR = Hazard Ratio; LVEF = Left Ventricular Ejection fraction; NS-VT = non sustained ventricular tachycardia.

The relation between NS-VT and mortality was not statistically significant also after excluding patients treated with antiarrhythmic medications (HR 0.59; 95% CI 0.30–1.18; p = 0.14).

We also assessed whether a “de novo” detection of NS-VT, defined as NS-VT occurring for the first time at the last pacemaker control, was associated with death. Among the 87 patients who had evidence of NS-VT at pace-maker interrogation during follow-up, a “de novo” NS-VT diagnosis was achieved in 24 patients (27.6%). At univariate Cox regression analysis “de novo” NS-VT showed a borderline association with death (p = 0.064, **Table 2**); however, the borderline association was lost at multivariable analysis (p = 0.38; **Table 3)**.

Of note, patients with a “de novo” NS-VT diagnosis did not differ from those with previous detection of NS-VT in terms of survival (p = 0.32) and causes of death (death from non-cardiovascular comorbidities, p = 0.81; death for acute heart failure, p = 0.22; death for infarct-related non-arrhythmic complications, p = 0.40).

## Discussion

The main finding of our study is that, in patients with implanted pacemaker due to atrioventricular block or sinus node dysfunction, asymptomatic episodes of NS-VT are not associated with an increased mortality. Our data show, in fact, that there was a non-significant trend to a lower mortality in patients with evidence of NS-VT on pacemaker controls, which might simply be related to case or, as an alternative, to closer clinical attention and surveillance of patients found to have NS-VT during pacemaker checks.Currently available pacemakers are capable of multiple functions and provide very useful information to physicians with regard to arrhythmias, patient activity and neuro-hormonal impairment and more. Part of this information has been proven to be useful in the prevention of adverse events, like stroke prevention in patients with prolonged episodes of atrial fibrillation accidentally discovered during routine pacemaker checks. Clinical implications or other forms of arrhythmias are less clear and should be properly addressed. In particular asymptomatic episodes of NS-VT are frequently discovered in patients with dual-chamber pacemakers and, at present, their clinical implications are unclear. Although some recent studies [13–16] have also reported a lack of prognostic impact of NS-VT detected at routine pacemaker checks, our study provides some new information to this regard.

Previous studies, indeed, enrolled heterogeneous populations, mixing patients with preserved and depressed LVEF, as well as patients enrolled after their first pacemaker implantation with patients undergoing battery replacement and patients with dual and single-chamber devices. Conversely, we selected a population of patients enrolled at the time of a first pacemaker implantation, without any history of previous ventricular arrhythmias and with preserved LVEF. Even in this accurately selected population, however, we failed to detect any significant negative impact of NS-VT on prognosis.

Of note, we found a borderline univariate association between de novo occurrence of NS-VT at the last pacemaker check and death. In this subset, a role of NS-VT as a marker of worsening of cardiovascular conditions deserves consideration. Since we cannot exclude that some patients may have experienced NS-VT before the following pacemaker check and died thereafter, association between de novo NS-VT occurrence and death may have been underestimated. Accordingly, our data suggest that a de novo diagnosis of NS-VT revealed by pacemaker interrogation should deserve a cardiovascular re-evaluation as compared to chronic (longer than six months) history of ventricular arrhythmias that has been confirmed not prognostically relevant as in previous studies [13–15].

In our study population, only a minority of patients investigated the presence of CAD as a possible explanation of NS-VT appearance. In these patients, evidence of myocardial ischemia was documented in 2 out of 13 subjects who underwent myocardial scintigraphy and a critical coronary artery stenosis was discovered in 4 out of 9 patients who underwent coronary angiography. Although it could be speculated from this small subset that only a minority of patients had CAD as a possible explanation of NS-VT occurrence, the etiology of NS-VT cannot be deduced from the results of our study. Of note, the prevalence of structural heart disease was similar between Groups 1 and 2, baseline pharmacological therapy did not differ between groups, as well as the rate of adverse cardiovascular events. A negative effect of pacing per se cannot be excluded as a cause of NS-VT occurrence. However, pacemaker programming was optimized in all patients in order to minimize right ventricular pacing and the incidence of NS-VT did not differ on the base of the indication to pacemaker implantation (atrioventricular block vs sinus node disease). In the age of magnetic resonance-conditional pacemakers, it would be interesting to investigate if ventricular arrhythmia appearance could be explained by de novo evidence of right or left ventricular myocardial abnormalities like edema or myocardial fibrosis.

## Limitations of the study

Some limitations of our study should be acknowledged. First, the sample size and the number of events might have been not large enough to have sufficient statistical power; accordingly, our data should be considered as explorative and needing confirmation in larger populations.

Second, we obtained data about pharmacological therapy and echocardiographic parameters only at enrollment. Thus, we cannot establish whether changes of these variables during follow-up might have influenced the relation of NS-VT with clinical outcome. However, prognostic studies are usually designed to assess the potential predictive variables at enrolment and we followed this consolidated practice.

Third, we cannot exclude that the use of beta-blockers might have blunted the negative effects of NS-VT on clinical outcome. However, the favorable effects of beta-blockers have been shown to be independent of NS-VT and in our population they were equally distributed in patients with and without NS-VT (Table 1).

Finally, it could have been useful to assess whether any differences in the effects on clinical outcome existed between monomorphic and polymorphic NS-VT. Unfortunately, however, this was not possible since NS-VTs were identified from PM interrogation and, therefore, the morphology of NS-VT could not be established.
